# Inflammatory Stress Sensitizes the Liver to Atorvastatin-Induced Injury in ApoE-/- Mice

**DOI:** 10.1371/journal.pone.0159512

**Published:** 2016-07-18

**Authors:** Wei Wu, Lei Zhao, Ping Yang, Wei Zhou, Beibei Li, John F. Moorhead, Zac Varghese, Xiong Z. Ruan, Yaxi Chen

**Affiliations:** 1 Center for Lipid Research & Key Laboratory of Molecular Biology for Infectious Diseases (Ministry of Education), Institute for Viral Hepatitis, Department of Infectious Diseases, the Second Affiliated Hospital, Chongqing Medical University, Chongqing, China; 2 The Collaborative Innovation Center for Diagnosis and Treatment of Infectious Diseases (CCID), Zhejiang University, Hangzhou, China; 3 John Moorhead Research Laboratory, Center for Nephrology, University College London Medical School, Royal Free Campus, University College London, London, United Kingdom; RWTH Aachen, GERMANY

## Abstract

Statins, which are revolutionized cholesterol-lowing agents, have been reported to have unfavorable effects on the liver. Inflammatory stress is a susceptibility factor for drug-induced liver injury. This study investigated whether inflammatory stress sensitized the liver to statin-induced toxicity in mice and explored the underlying mechanisms. We used casein injection in ApoE-/- mice to induce inflammatory stress. Half of the mice were orally administered atorvastatin (10mg/kg/d) for 8 weeks. The results showed that casein injection increased the levels of serum pro-inflammatory cytokines (IL-6 and TNFα). Atorvastatin treatment increased serum alanine aminotransferase (ALT) and aspartate aminotransferase (AST) in casein injection mice. Moreover, atorvastatin treatment exacerbated hepatic steatosis, inflammation and fibrosis, as well as increased hepatic reactive oxygen species (ROS) and malondialdehyde in casein injection mice. However, above changes were not observed in atorvastatin treated alone mice. The protein expression of liver nuclear factor erythroid 2-related factor 2 (Nrf2) and the mRNA expressions of Nrf2 target genes were increased, together with the enhancement of activities of hepatic catalase and superoxide dismutase in atorvastatin treated alone mice, but these antioxidant responses were lost in mice treated with atorvastatin under inflammatory stress. This study demonstrates that atorvastatin exacerbates the liver injury under inflammatory stress, which may be associated with the loss of adaptive antioxidant response mediated by Nrf2.

## Introduction

Hypercholesterolemia, characterized by a high level of circulating low-density lipoprotein cholesterol (LDL-C), is a crucial risk factor for cardiovascular disease (CVD)[1]. Statins are a class of drugs which exhibit a powerful hypocholesterolemic effect by inhibiting the activity of 3-hydroxy-3-methylglutaryl coenzyme A reductase (HMG-CoAR). Treatment with statins has been reported to markedly reduce morbidities and mortalities of major cardiovascular events in patients[2, 3].

The liver is the primary organ responsible for the metabolism and action of statins, therefore, hepatoxicity induced by statins is a matter of concern for physicians. Mild elevations of serum transaminases are observed in approximately 1–3% of patients on statins[4], while in most cases the elevation of liver enzymes is self-limiting, and only 3% of these patients undergo a subsequent persistent elevation of greater than three times the upper limit of normal[5], indicating that most patients can adapt to statins, and only a small fraction of patients are sensitive to statin-induced liver injury. However, the risk factors and mechanism of statin-induced liver injury are not fully understood.

Oxidative stress has been identified to involve in hepatotoxicity induced by many drugs[6], but the development and severity of liver damage rely on the battle between drug-induced hazardous stress and adaptive responses of hepatocytes[7]. Derick Han proposed that[8], for a majority of patients, the activation of adaptation pathways such as nuclear factor erythroid 2-related factor 2 (Nrf2), a key regulator of the antioxidant defense, helps hepatocytes adapt to the drug, and no liver injury occurs. While in a minority of individuals, some genetic or environmental risk factors impair the adaptation pathways, which may sensitize the liver to injury caused by some hepatotoxic drugs. Recently, some researches showed that the activation of Nrf2 was impaired in some chronic inflammatory diseases, such as chronic granulomatous disease[9], asthma[10] and chronic tubulo-interstitial nephropathy[11]. On the other hand, inflammatory stress has been considered as a determinant of susceptibility for drug induced liver injury[12–14]. Therefore, it is reasonable to assume that inflammatory stress may sensitize the liver to drug induced injury via disrupting Nrf2-mediated adaptive response. Whether this hypothesis can explain the response of the liver to statins is unclear.

Metabolic disorders, such as hypercholesterolemia, atherosclerosis, obesity, and diabetes, are closely associated with metabolic inflammation, which is a chronic low-grade systemic inflammation characterized by abnormal cytokine production, increased acute-phase reactants and other mediators, and activation of a network of inflammatory signalling pathways[15]. Our previous studies demonstrated that chronic low-grade systemic inflammation could disrupt HMG-CoAR mediated cholesterol synthesis, resulting in intracellular lipid accumulation and statin resistance[16]. A consequence of inflammatory stress is that statin resistance necessitates high doses of statins to protect cardiovascular from cholesterol accumulation[16], but it may be an additional burden on the liver for drug metabolism. Therefore, it is of clinical importance to investigate the effect of statins on the liver under chronic inflammatory stress.

In the present study, ApoE-/- mice fed with a western diet were employed to mimic patients with hypercholesteremia, and some mice were injected with casein subcutaneously to establish chronic systemic inflammation. The purpose of this study was to investigate whether mice with chronic inflammation are prone to statin-induced liver injury and explore the underlying mechanisms.

## Methods

### Animal model

The Animal Care Committee of Chongqing Medical University approved all the procedures. Six to eight-week-old male ApoE-/- mice (C57BL/6J background) were obtained from the Experimental Animal Center, Chongqing Medical University. All animals were kept in sterile cages at a temperature of 21±2°C with a 12h light/dark cycle, and were provided with food and water *ad libitum*. After 7 days of acclimatization, the mice were fed a western diet (D12079B, Research Diets Inc., New Brunswick, NJ, USA) and randomly assigned to subcutaneous injections of 0.5ml saline or 0.5ml 10% casein every other day. Atorvastatin (Pfizer) (10mg/kg/day) were orally administered to half of the mice injected with saline or 10% casein. The protocol for early/humane endpoints in cases where animals became severely ill prior to the experimental endpoint was implemented according to the animal facility rules. If the animals are moribund or in a state of impending death, they should be immediately euthanized. The animals were monitored daily, and none mouse died nor exhibited clinical signs of suffering, distress or pain during the experimental procedure. All mice were fasted overnight, then were sacrificed under anesthesia by intraperitoneal injection of pentobarbital sodium (60mg/kg body weight) at 8 weeks after above treatments.

### Serum analysis

Serum cytokine levels were determined using an enzyme-linked immune sorbent assay (ELISA) kit (Millipore, Billerica, MA, USA). Serum levels of total cholesterol (TC), low density lipoprotein cholesterol (LDL-C), triglyceride (TG), alanine transaminase (ALT) and aspartate aminotransferase (AST) were measured by an automatic biochemistry analyzer. Serum free fatty acid (FFA) concentrations were determined by an ELISA kit (MLBIO Biotechnology Co.Ltd, Shanghai, China).

### Histopathological analysis

Sections of paraffin-embedded liver samples were used for H&E (Haematoxylin and Eosin), Sirius red and immunohistochemistry staining. The immunohistochemistry procedure was followed instructions of the commercial kit (ZsBio, Beijing, China). The primary antibody Biotin anti-mouse F4/80 (BM8, #123105) and anti-Nrf2 (C-20, #sc-722) were purchased from Biolegend (San Diego, CA, USA) and Santa Cruz Biotechnology (Dallas, Texas, USA) respectively. The Sirius red positive areas were quantified using Image J software in 5 separated fields from different mice of each group. Sections of frozen liver samples were stained with Oil Red O using standard techniques.

### Quantitative measurements of hepatic triglyceride (TG), free fatty acid (FFA) and total cholesterol (TC) levels

Hepatic lipids were extracted in chloroform/methanol (2:1). TG and FFA levels were quantified using an enzymatic assay kit (DONGOU, Wenzhou, Zhejiang, China) and an ELISA kit (MLBIO Biotechnology Co. Ltd, Shanghai, China) respectively. The procedures were conducted according to the manufacturer’s instructions. TC levels were determined by enzymatic assays as described previously[17]. The concentrations of TG, FFA and TC were normalized to the weight of wet liver.

### Real-time PCR

Total RNA were extracted from liver tissue homogenates using the Trizol reagent (Takara Life Technologies, Carlsbad, Janpan). Real-Time reverse transcription polymerase chain reaction (PCR) was performed in a Bio-Rad Sequence Detection System (Hercules, US) using SYBR Green dye (Applied Biosystems Inc, Foster City, US) in accordance with the manufacturer’s instructions. β-actin served as an internal control gene for data normalization. The sequences of primers for PCR are listed in Table 1.

**Table 1:** Mouse primers for real-time polymerase chain reaction.

Genes	Mouse primers	
FAS	Forward	5’-CCTGGATAGCATTCCGAACCT-3’
	Reverse	5’-AGCACATCTCGAAGGCTACACA-3’
ACC	Forward	5’-CGCTCAGGTCACCAAAAAGAAT-3’
	Reverse	5’-GTCCCGGCCACATAACTGAT-3’
SREBP1	Forward	5’-GCCCACAATGCCATTGAGA-3’
	Reverse	5’-CAGGTCTTTGAGCTCCACAATCT-3’
IL-1β	Forward	5’-GCTTCAGGCAGGCAGTAT-3’
	Reverse	5’-ACAAACCGCTTTTCCATCT-3’
TNFα	Forward	5’-CAGCCGATGGGTTGTACCTT-3’
	Reverse	5’-GGCAGCCTTGTCCCTTGA-3’
MCP1	Forward	5’-GTCTGTGCTGACCCCAAGAAG-3’
	Reverse	5’-TGGTTCCGATCCAGGTTTTTA-3’
αSMA	Forward	5’-CCAGAGCAAGAGAGGGATCCT-3’
	Reverse	5’-TGTCGTCCCAGTTGGTGA-3’
COL4	Forward	5’-CCGAGCCAGTCCATTTATAGAATG-3’
	Reverse	5’-CAGCGAAGCCAGCCAGAA-3’
TGFβ	Forward	5’-GCAGTGGCTGAACCAAGGA-3’
	Reverse	5’-AGCAGTGAGCGCTGAATCG-3’
HO-1	Forward	5’-AACAAGCAGAACCCAGTCTATGC-3’
	Reverse	5’-AGGTAGCGGGTATATGCGTGGGCC-3’
SOD2	Forward	5’-CAGACCTGCCTTACGACTATGG-3’
	Reverse	5’-CTCGGTGGCGTTGAGATTGTT-3’
NQO1	Forward	5’-TGGCCGAACACAAGAAGCTG-3’
	Reverse	5’-GCTACGAGCACTCTCTCAAACC-3’
β-actin	Forward	5’-CGATGCCCTGAGGCTCTTT-3’
	Reverse	5’-TGGATGCCACAGGATTCCAT-3’

### Western blot

Total proteins from liver homogenates were extracted using RIPA buffer. Nucleus and cytoplasm proteins were prepared using the extraction buffer as previously described[18]. Identical amounts of proteins were subjected to SDS-PAGE, and then transferred to PVDF membranes. The membranes were blocked with 5% milk, and then incubated with following primary antibodies: FAS (H-300, #sc-20140), ACCα (H-76, #sc-30212), SREBP1 (H-160, #sc-8984), COL4A2 (T-15, #sc-70246), TGFβ1 (V, #sc-146), MCP1 (FL-148, #sc-28879) and Nrf2 (C-20, #sc-722) from Santan Cruz Biotechnology, USA; IL-1β (#16806-1-AP), TNFα (#60291-1-Ig) and αSMA (#14395-1-AP) from Proteintech, USA; Histone H3 (T22, #BS1405) from Bioworld Technology, USA; β-actin (1E9A3, #TA-09) from ZSGB-BIO, China. Thereafter, the membranes were incubated with corresponding secondary peroxidase-coupled antibody. Finally, the detection procedure was performed using Immobilon Western Chemiluminescent HRP Substrate (Millipore, Temecula, CA, USA). Densitometric analysis was performed using the Image J software. β-actin served as a loading control for total or cytosolic proteins, and Histone H3 served as the loading control for nuclear proteins.

### Evaluation of the superoxide anion (*O*_*2*_^*-*^) levels in the liver

Hepatic *O*_*2*_^*-*^ levels were measured with the oxidative fluorescent dye dihydroethidium (DHE). Briefly, liver cryosections (10um) were incubated with 10uM DHE in the dark for 30 min at 37°C, washed three times and examined with a fluorescence microscope immediately.

### Determination of hydrogen peroxide (H_2_O_2_) and malondialdehyde (MDA) levels

Measurements of hepatic H_2_O_2_ and MDA content were performed using commercial kits (Beyotime, China) following instructions from the manufacturers. H_2_O_2_ and MDA concentrations were normalized by total liver proteins.

### Antioxidant enzyme activity assays

Homogenates of liver tissues were centrifuged at 12000g for 10 min at 4°C. The supernatants were collected for catalase (CAT) activity and total superoxide dismutase (SOD) activity assays using commercial kits (Beyotime, China). The enzyme activity was expressed as U/mg protein in the homogenates.

### Statistical analysis

The data are presented as the mean ± SD. Comparisons among groups were determined with one-way analysis of variance followed by Tukey’s posttest using GraphPad Prism5 software. A *P* value less than 0.05 was considered significant.

## Results

### Effect of atorvastatin on serum parameters of ApoE-/- mice in the absence or presence of casein injection

A chronic low-grade systemic inflammation was induced in ApoE-/- mice using subcutaneous injection of 10% casein on alternate days for 8 weeks. Serum levels of pro-inflammatory cytokines (IL-6 and TNFα) were significant increased in mice with casein injection compared to those without casein injection (Table 2), suggesting that chronic inflammation was successfully induced in ApoE-/- mice. Serum LDL-C levels were significantly decreased in casein injected mice compared to controls (Table 2), which is consistent with our previous studies[19]. Atorvastatin reduced serum TC and LDL-C levels significantly, but had no effects on serum TG and FFA levels (Table 2). Moreover, we assessed liver function of mice by detecting serum ALT and AST levels, and the result showed that only casein+atorvastatin mice exhibited a significant increase in the serum levels of ALT and AST (Table 2).

**Table 2:** Effect of atorvastatin on serum parameters of ApoE-/- mice with or without casein injection.

	control	atorvastatin	casein	casein+atorvastatin
IL6 (pg/ml)	11.54±4.36	10.13±2.60	33.93±7.28*	30.91±7.82*
TNFα (pg/ml)	4.08±1.87	2.66±0.99	84.43±39.47*	80.65±22.99*
TC(mmol/L)	30.47±2.20	22.14±3.07*	19.51±1.64*	15.23±1.23#
LDL-C(mmol/L)	5.83±0.58	4.09±0.96*	3.33±0.51*	2.11±0.24#
TG(mmol/L)	1.14±0.35	1.18±0.17	0.69±0.04*	1.02±0.08
FFA(umol/L)	55.73±20.04	60.16±11.97	63.70±10.62	40.70±5.52
ALT(U/L)	21.50±6.61	24.00±4.90	26.80±7.29	43.00±8.87*#
AST(U/L)	94.67±23.79	93.20±29.28	115.00±36.97	257.50±105.04*#

IL6, interleukin6; TNFα, tumor necrosis factor α; TC, total cholesterol; LDL-C, low-density lipoprotein cholesterol; TG, total triglyceride; FFA, free fatty acid; ALT, alanine aminotransferase; AST, aspartate aminotransferase.

The serum levels of cytokines, lipids and liver function in ApoE-/- mice after the 8-week experimental period. Results are expressed as mean ± SD (n = 6)

*P<0.05 versus control

#*P*<0.05 versus casein injected alone group.

### Atorvastatin exacerbates hepatic steatosis in ApoE-/- mice under inflammatory stress

**Fig 1:**
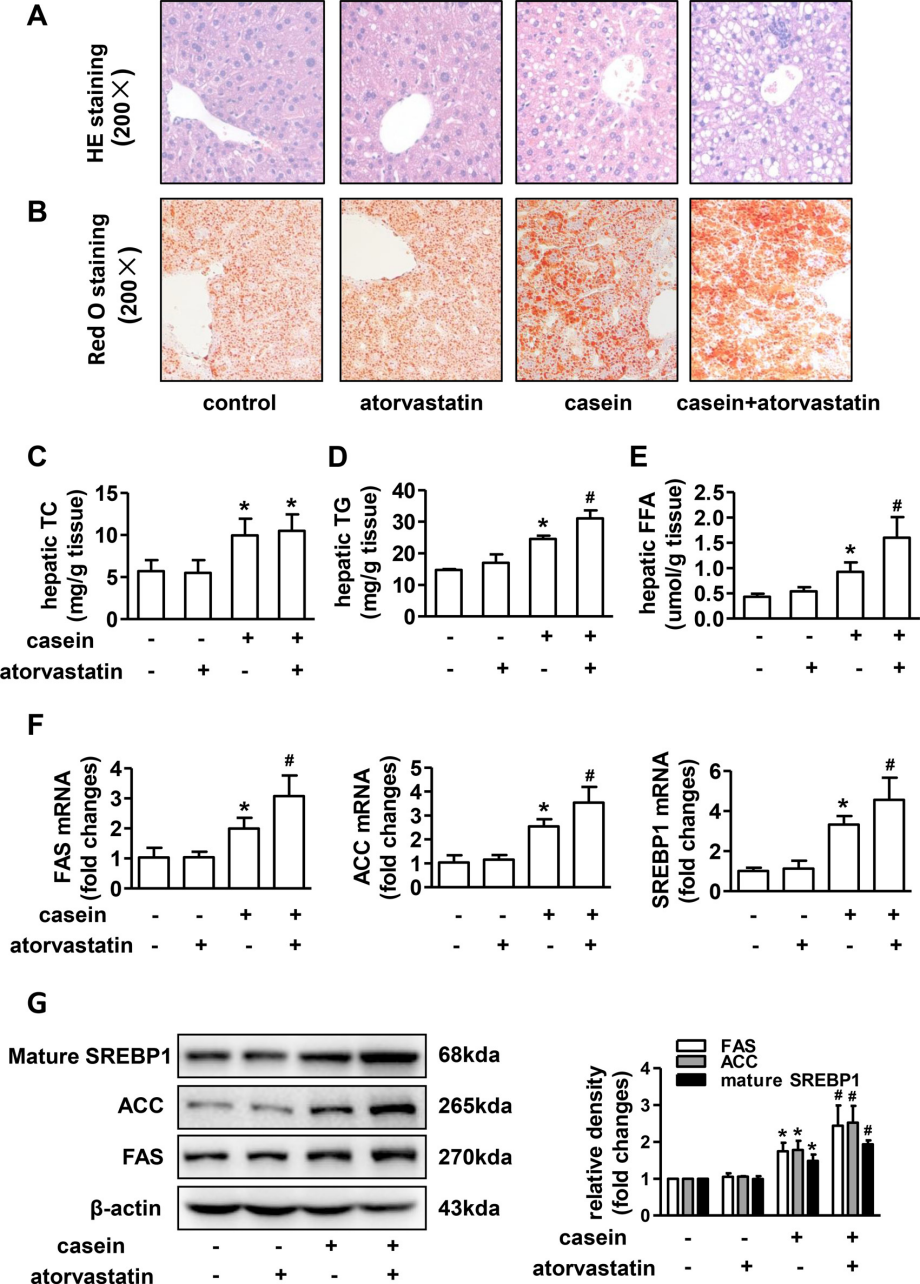
Effect of atorvastatin on lipid accumulation in livers of ApoE-/- mice. Liver sections of representative mice from each group were stained with (A) H&E or (B) Oil Red O. The contents of liver (C) TC, (D) TG and (E) FFA were quantified as described in methods (n = 6). (F) The mRNA expressions of lipogenic genes in livers of ApoE-/- mice (n = 6). (G) The protein expressions of lipogenic genes in livers of ApoE-/- mice (n = 3). The histogram represents the densitometric scans for target protein bands normalized by β-actin and expressed as fold changes relative to protein expression in control mice. The results are depicted as mean ± SD, *P<0.05 versus control group, #*P*<0.05 versus casein injected alone group.

Hematoxylin and eosin (H&E) staining showed no apparent changes of liver histology manifestation in atorvastatin treated alone mice compared with controls, marked vacuolar degeneration of the liver was observed in casein injected alone mice, and the vacuolar degeneration was further aggravated in casein+atorvastatin mice(Fig 1A). Oil Red O staining revealed that the number of lipid droplets obviously increased in casein injected alone mice compared with controls, and the lipid droplets accumulation was further accentuated in casein+atorvastatin mice (Fig 1B). Quantitative analysis of intrahepatic lipids showed that TC, TG and FFA concentrations were not significantly changed in atorvastatin treated alone mice compared with controls, but TG and FFA contents were significantly increased in atorvastatin treated mice under inflammatory stress (Fig 1C–1E).

Because the exacerbation of hepatic steatosis was mainly attributable to an accumulation of TG and FFA, we assessed the mRNA and protein expressions of lipogenic genes in livers of mice. In the non-inflamed condition, the mRNA and protein expressions of sterol regulatory element-binding protein1 (SREBP1), fatty acid synthase (FAS) and Acetyl-CoA carboxylase (ACC) were not significantly changed in atorvastatin treated mice (Fig 1F and 1G). Compared to the control group, casein injection significantly upregulated hepatic SREBP1, FAS and ACC mRNA and protein expressions, and casein plus atorvastatin further increased mRNA and protein expressions of these lipogenic genes(Fig 1F and 1G).

### Atorvastatin exacerbates the inflammatory injury and fibrosis in livers of ApoE-/- mice under inflammatory stress

**Fig 2:**
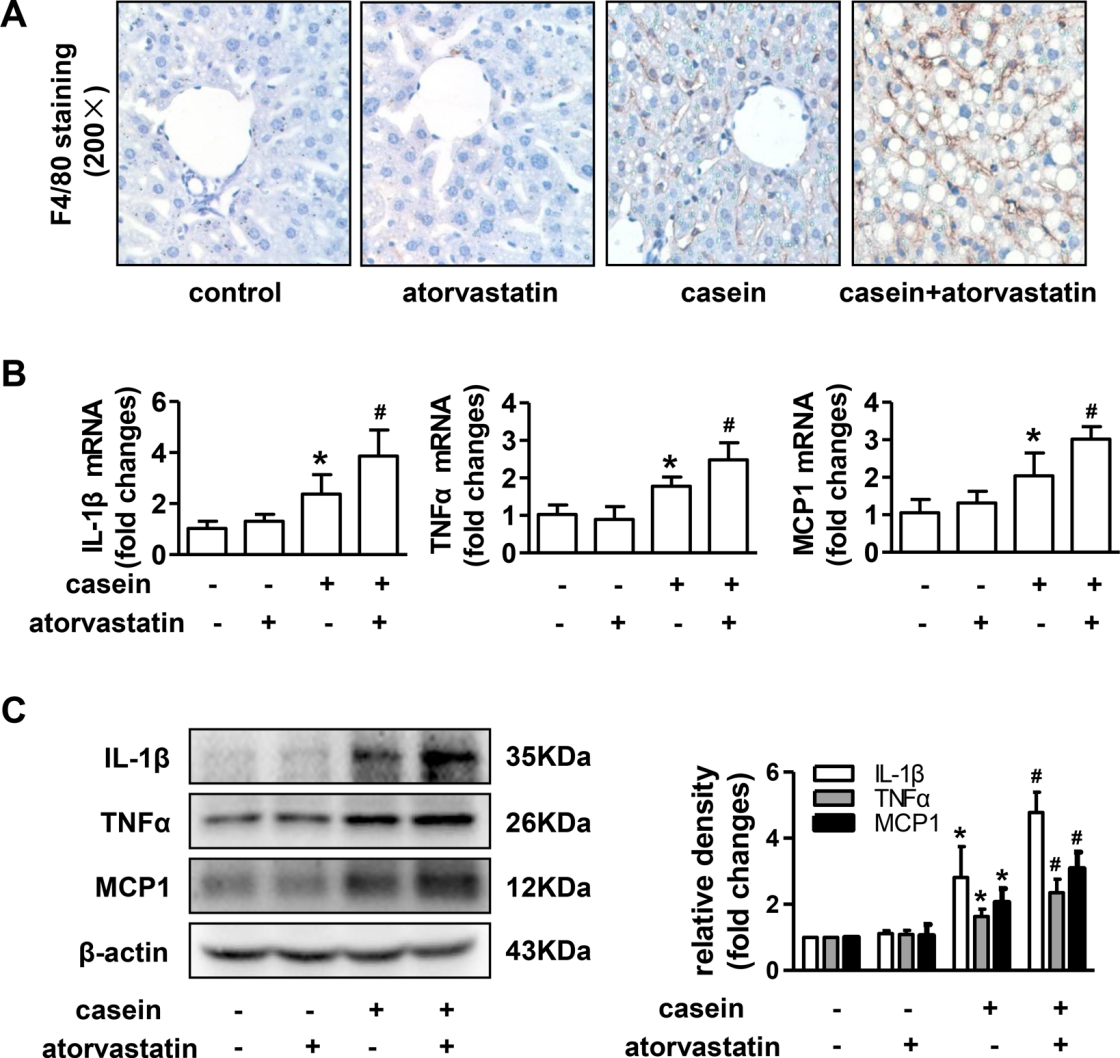
Effect of atorvastatin on hepatic inflammatory injury in ApoE-/- mice. (A) F4/80 immunohistochemical staining of liver sections from ApoE-/- mice. (B) The mRNA expressions of IL-1β, TNFα and MCP1 in livers of ApoE-/- mice (n = 6). (C) The protein expressions of IL-1β, TNFα and MCP1 in livers of ApoE-/- mice (n = 3). The histogram represents the densitometric scans for target protein bands normalized by β-actin and expressed as fold changes relative to protein expression in control mice. The results are depicted as mean ± SD, *P<0.05 versus control group, #*P*<0.05 versus casein injected alone group.

Results from liver immunohistochemical staining showed that the number of F4/80-positive macrophages was markedly increased in livers of casein injected alone mice compared with control mice, and the number was further increased in livers of casein+atorvastatin mice (Fig 2A). Compared with controls, the mRNA and protein expressions of hepatic interleukin-1β (IL-1β), monocyte chemoattractant protein-1 (MCP1) and tumor necrosis factorα (TNFα) were significantly increased in casein injected alone mice, and were further increased in casein+atorvastain mice (Fig 2B and 2C).

**Fig 3:**
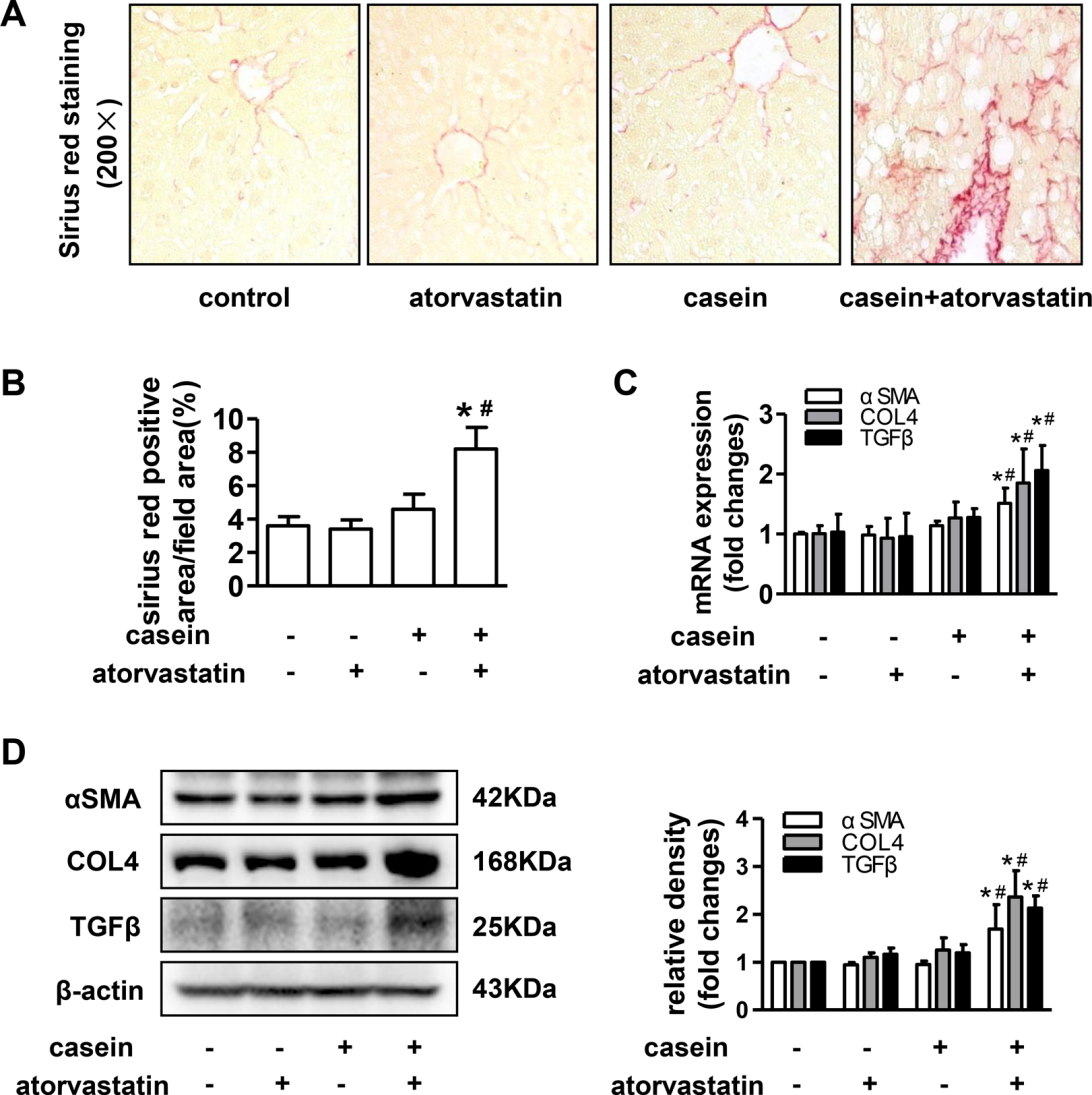
Effect of atorvastatin on hepatic fibrosis in ApoE-/- mice. (A) Sirius red staining of liver sections from ApoE-/- mice. (B) Quantitative analysis of Sirius red positive areas in separated fields from each group (n = 5). (C) The mRNA expression of αSMA, COL4 and TGFβ in livers of ApoE-/- mice (n = 6). (D) The protein expressions of αSMA, COL4 and TGFβ in livers of ApoE-/- mice (n = 3). The histogram represents the densitometric scans for target protein bands normalized by β-actin and expressed as fold changes relative to protein expression in control mice. The results are depicted as mean ± SD, *P<0.05 versus control group, #*P*<0.05 versus casein injected alone group.

Sirius red staining of liver sections indicated more prominent collagen fibres in casein+atorvastatin mice than that in other mice (Fig 3A and 3B). As predicted from the results of sirius red staining, the mRNA and protein expressions of α-smooth muscle actin (αSMA), collagen, type IV (COL4) and transforming growth factorβ (TGFβ) were markedly increased in casein+atorvastatin mice compared with those in control and casein injection alone mice (Fig 3C and 3D). Taken together, casein injection alone promoted the progression of steatosis into steatohepatitis, and atorvastatin accelerated the development of steatohepatitis under inflammatory stress.

### Atorvastatin promotes oxidative stress in livers of ApoE-/- mice under inflammatory stress

**Fig 4:**
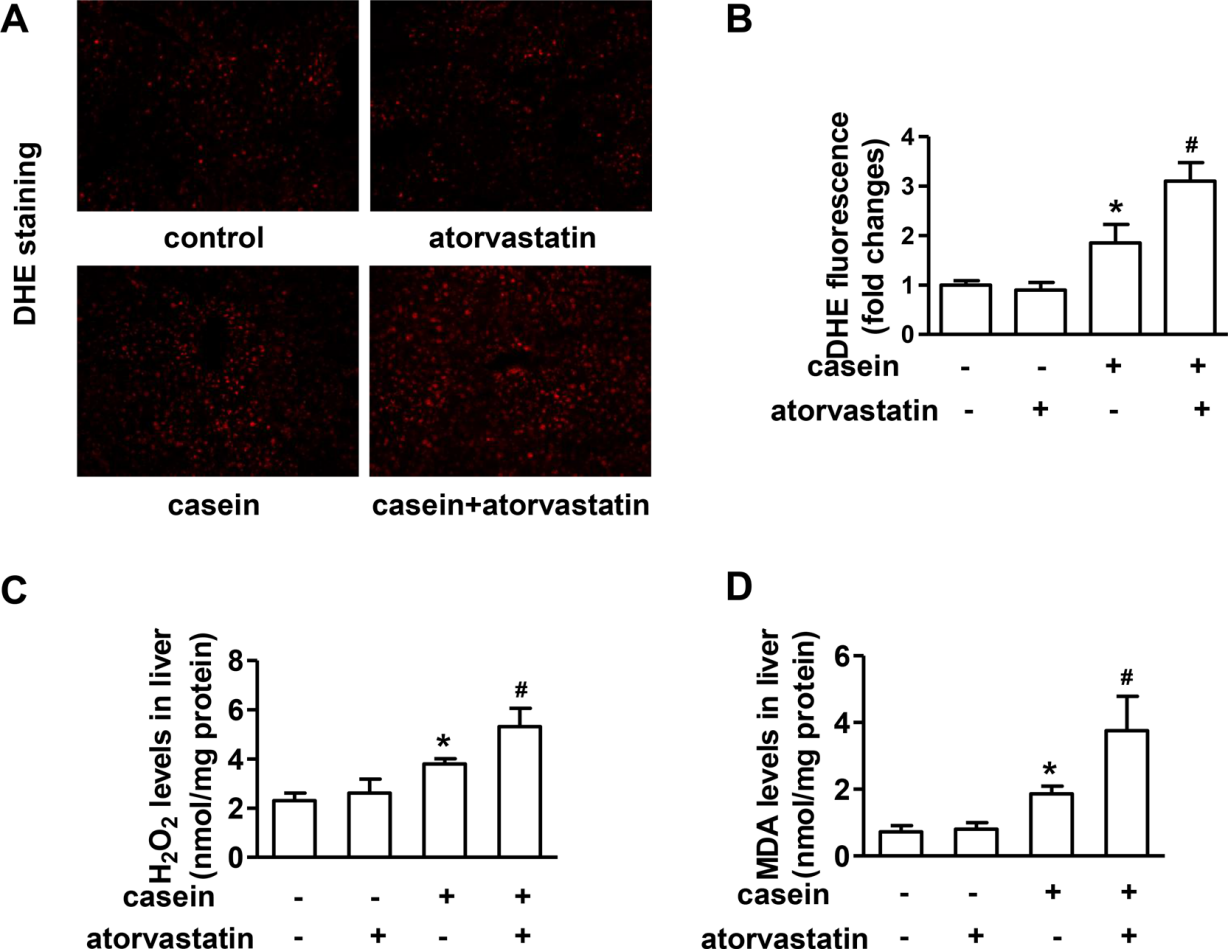
Effect of atorvastatin on the oxidative stress in livers of ApoE-/- mice. (A) Superoxide anion (*O*_*2*_^*-*^) accumulation in liver cryosection analyzed by DHE staining (original magnification ×200). (B) Fluorescence intensity analysis of DHE staining. Values were expressed as the folds of control (n = 4). (C) Hepatic H_2_O_2_ levels (n = 4). (D) Hepatic MDA levels (n = 4). Data were expressed as mean ± SD. *P<0.05 versus control group, #*P*<0.05 versus casein injected alone group.

To determine whether the deterioration of liver damage in mice treated with atorvastatin plus casein injection was associated with enhanced oxidative stress, we assessed the liver *O*_*2*_^-^ accumulation, hepatic H_2_O_2_ and MDA levels. As the results showed by DHE staining, there were no significant changes of liver *O*_*2*_^-^ accumulation between atorvastatin treated alone mice and controls, but casein injected alone induced a remarkable increase of liver *O*_*2*_^-^ accumulation, which was further increased by atorvastatin treatment (Fig 4A and 4B). The changes of hepatic H_2_O_2_ and MDA levels were consistent with the changes of liver *O*_*2*_^-^ accumulation (Fig 4C and 4D).

### The adaptive antioxidant response mediated by Nrf2 was lost in mice treated with atorvastatin under inflammatory stress

**Fig 5:**
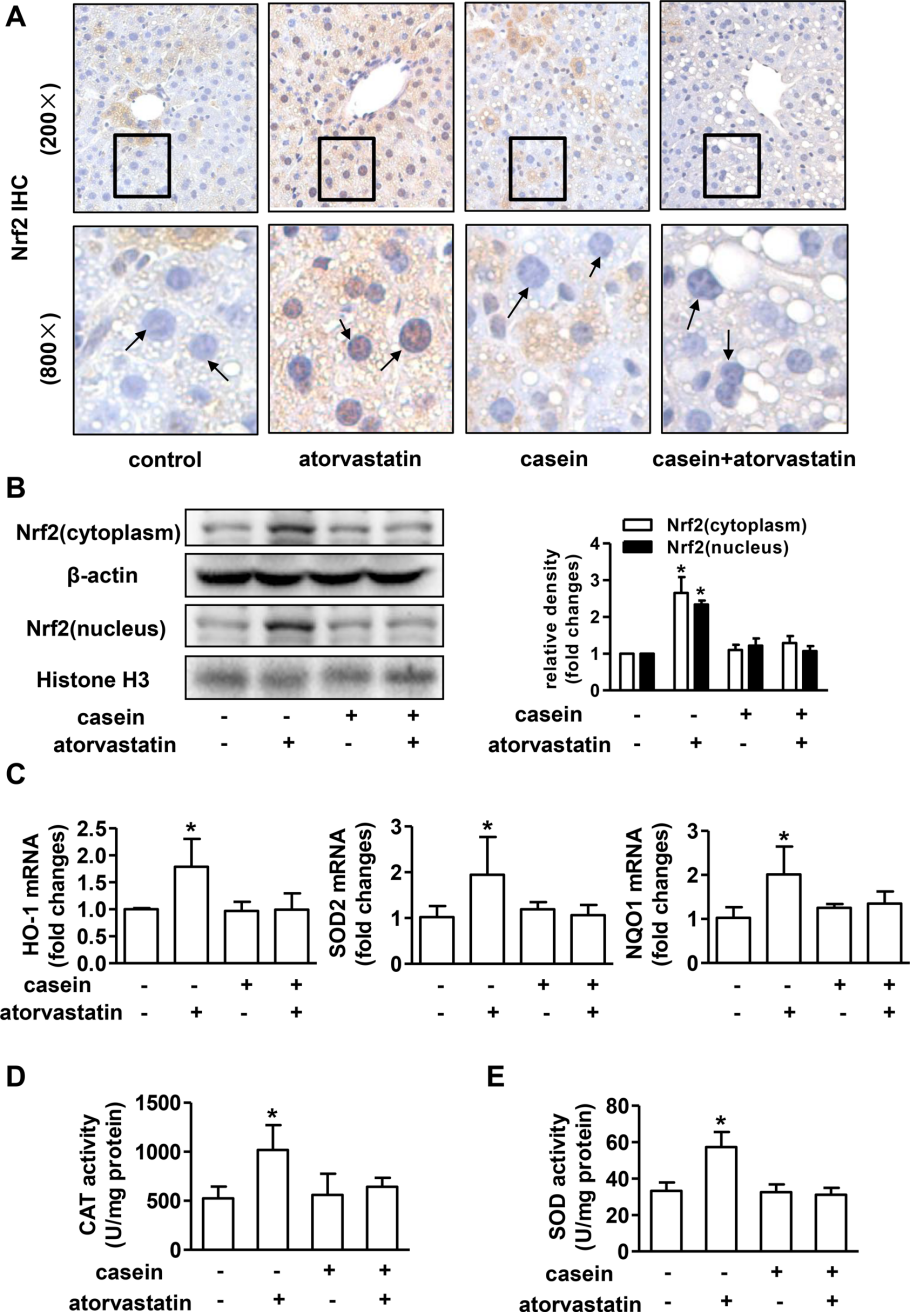
Adaptive antioxidant response mediated by Nrf2 in livers of ApoE-/- mice after atorvastatin treatment. (A) Immunohistochemical staining of Nrf2 in liver sections from ApoE-/- mice. (B) The protein expressions of Nrf2 in nucleus and cytoplasm fractions in livers of ApoE-/- mice (n = 3). (C) The mRNA expressions of HO-1, SOD2 and NQO1 in livers of ApoE-/- mice (n = 6). The enzymatic activity of (D) CAT and (E) SOD in livers of ApoE-/- mice (n = 4). The results are depicted as mean ± SD, *P<0.05 versus control group, #*P*<0.05 versus casein injected alone group.

Oxidative stress occurs when the pro-oxidative capacity overwhelms the antioxidant capacity, and the latter is primarily controlled by Nrf2[20]. To address whether the distinct response to atorvastatin induced oxidative injury in different metabolic condition (with or without chronic inflammation) depend on Nrf2-mediated antioxidant response. We evaluated the expression of Nrf2 in livers. Immunohistochemical examination revealed that positive staining of Nrf2 both in the nucleus and cytoplasm of hepatocytes was increased in mice treated with atorvastatin in non-inflamed condition, but not in mice injected with casein regardless of atorvastatin treatment (Fig 5A). Next, we examined the protein expression of Nrf2 in the nucleus and cytoplasm extract of the liver, and the result was in consistent with the Immunohistochemical staining (Fig 5B). The changes of mRNA expressions of Nrf2 target gene heme oxygenase-1 (HO-1), manganese superoxide dismutase (SOD2) and NAD(P)H:quinine oxidoreductase (NQO1) were similar with the changes of Nrf2 protein levels in nuclear fractions (Fig 5C).

Superoxide dismutase (SOD) and catalase (CAT) have the ability of scavenging superoxide anion and hydrogen peroxide respectively[21, 22], so we further examined activities of SOD and CAT. The results showed that hepatic SOD and CAT activities were apparently enhanced upon atorvastatin treatment in non-inflamed condition, but not in inflamed condition (Fig 5D and 5E).

## Discussion

Metabolic inflammation has been identified to participate in the pathogenesis of many metabolic disorders, such as obesity, diabetes and cardiovascular disease[15]. Because metabolic inflammation is a chronic sterile inflammation and casein as a major component of cow milk proteins can trigger a non-infectious systemic inflammatory response via stimulating B-lymphocytes[23, 24], we used casein injection to establish the inflammation model in this study. Serum IL6 and TNFα levels were remarkably increased after casein injection for 8 weeks, suggesting that a chronic systemic inflammation was successfully induced in ApoE-/- mice.

Next, to determine whether inflammation sensitized the liver to atorvastatin induced injury, we assessed the effect of atorvastatin on livers of mice with or without inflammation. The results showed that atorvastatin had no adverse effect on the liver of mice in non-inflamed condition. However, under inflammatory stress, atorvastatin elevated serum ALT and AST levels, as well as exacerbated hepatic steatosis, inflammation and fibrosis, indicating that atorvastatin promoted the development of steatohepatitis under inflammatory stress.

Although the mechanisms responsible for drug-induced liver injury have not been fully clarified, oxidative stress due to overproduction of reactive oxygen species (ROS) plays an important role in this process[25–27]. On the one hand, ROS can promote hepatocyte fat accumulation through activating SREBP1c and fatty acid synthesis[28, 29]. On the other hand, excess ROS can initiate lipid peroxidation, leading to the formation of reactive aldehyde, such as malondialdehyde (MDA)[30]. Both ROS and lipid peroxidation products can trigger the production of many cytokines such as TNFα, IL-1β and TGFβ that favoring inflammation and fibrosis[31–33]. In this study, we observed that atorvastatin aggravated liver fat accumulation under inflammatory stress, characterized by increased hepatic TG and FFA, and upregulated expressions of lipogenic genes including FAS, ACC and SREBP1. Moreover, the mRNA and protein expressions of cytokines/chemokines and genes of fibrosis markers were also increased in livers of mice treated with atorvastatin under inflammatory stress, suggesting that the liver injury induced by atorvastatin may be associated with oxidative stress. Thus we further evaluated the levels of ROS and MDA in livers of mice, and found that the contents of ROS and MDA were paralleled with the liver injury, indicating that oxidative stress, at least partly, involved in the exacerbation of liver damage in mice treated with atorvastatin under inflammatory stress.

However, why oxidative stress injury only occurred in livers of mice treated with atorvastatin in inflamed condition, but not in non-inflamed condition. Jamal Bouitbir demonstrated that atorvastatin induced oxidative stress only in glycolytic skeletal muscles, but not in oxidative muscles, which had a higher antioxidant capacity[34]. Moreover, atorvastatin initially increased ROS production, which subsequently stimulated the antioxidant capacity to maintain the homeostasis in cardiac muscles, whereas the failure to activate antioxidant capacity leaded to the toxic impairment in glycolytic skeletal muscles[35]. These findings suggest that the antioxidant capacity determines the response of organs to statin-induced oxidative injury. So we evaluated the primary antioxidant defense in livers of mice.

Nrf2 is the master regulator of the genes encoding many antioxidant and phase II detoxifying enzymes, such as NQO1, SOD, HO-1, and catalase[36, 37]. Under basal conditions, Nrf2 is tethered in the cytoplasm by kelch-like ECH associating protein 1 (Keap1). When exposure to oxidative stress, Nrf2 escapes from Keap1, translocates into the nucleus, and induces a battery of cytoprotective genes as adaptive responses[20]. It has been demonstrated that Nrf2 plays an important role in the protection against drug induced hepatotoxicity[20, 36]. Nrf2-/- mice were more susceptible to drug-induced hepatotoxicity[38, 39]. Whereas activation of Nrf2 protected against the hepatotoxicity induced by various hepatotoxicants, such as acetaminophen (APAP), carbon tetrachloride and cadmium[40]. In addition, several evidences showed that loss of Nrf2 resulted in greater induction of lipogenic genes and progression of steatohepatitis in mice fed a high-fat diet or MCD diet[41–43], suggesting that Nrf2 may play a negative role in hepatic lipid modulation. In this study, Nrf2 signaling pathway was activated in livers of mice after atorvastatin treatment in non-inflamed condition. However, this adaptive response was lost in livers of mice under inflammatory stress, which may contribute to the upregulation of lipogenic genes including FAS, ACC and SREBP1, and the accelerated progression of steatohepatitis.

In conclusion, chronic inflammation sensitized the liver to atorvastatin induced injury via disturbing the activation of Nrf2-mediated adaptive antioxidant response. Therefore, it may be necessary for physicians to pay more attention to the liver adverse effects of statins in patients with chronic inflammation.
